# Efficient In/SSZ-39 catalysts for the selective catalytic reduction of NO with CH_4_

**DOI:** 10.3389/fchem.2024.1439581

**Published:** 2024-07-26

**Authors:** Sufeng An, Peng Wang, Kuanling Wang, Xuehai Wang, Baozhong Li, Xinwen Guo

**Affiliations:** ^1^ SINOPEC (Dalian) Research Institute of Petroleum and Petrochemicals, Dalian, China; ^2^ Dalian University of Technology, Chemical Engineering Institute, Dalian, China

**Keywords:** CH_4_-SCR, In/SSZ-39, highly dispersed InOx species, low temperature activation of CH_4_, high CH_4_ selectivity

## Abstract

The M/SSZ-39 catalysts (M = In, Co, Cu, Fe) with different metal species and metal loadings were synthesized using the wet impregnation method on a small-pore SSZ-39 molecular sieve. X-ray diffraction (XRD), transmission electron microscopy (TEM), nitrogen adsorption-dehydrogenation and hydrogen temperature program reduction (H_2_-TPR) were employed to characterize the effects of various metal components and metal loadings on the performance of CH_4_ selective catalytic reduction of NO reaction (CH_4_-SCR). The characterization results showed that the In/SSZ-39 catalyst exhibited significantly higher catalytic activity compared to the Cu-, Co-, and Fe/SSZ-39 catalysts, suggesting that indium (In) is a more suitable active ingredient for the CH_4_-SCR reaction. The xIn/SSZ-39 (x = 1, 2, 3, x represents the In loadings of 1.0 wt%, 2.0 wt% and 3.0 wt%) catalysts, with different In loadings, all present excellent CH_4_-SCR performance. By varying the In loadings, the type of In species present in the catalyst can be regulated, thus enhancing DeNOx activity and CH_4_ selectivity in the CH_4_-SCR reaction. At a low temperature of 400 °C and a low CH_4_/NO feed ratio (CH_4_/NO = 1), the 3In/SSZ-39 catalyst, featuring highly active InOx clusters, achieves the best low-temperature CH_4_-SCR performance, with a high NO conversion rate of up to 90% and a CH_4_ selectivity of up to 74.2%.

## 1 Introduction

Nitrogen oxides (NOx) are major pollutants that not only directly jeopardize human health but also trigger a series of environmental issues such as acid rain, photochemical smog, ozone hole, etc (Wang et al., 2017). Thus, addressing the pressing requirements for NOx emission reduction (DeNO_x_) is crucial for the sustainable development of human society. One of the primary sources of NOx emissions is industrial flue gas from thermal power plants, cement plants, and petrochemical facilities. The main method for reducing NO emissions is ammonia selective catalytic reduction (NH_3_-SCR), which uses V_2_O_5_-WO_3_ (MoO_3_)/TiO_2_ as the catalyst (Han et al., 2019; Wang et al., 2021). However, vanadium catalysts are biological toxicity, and using NH_3_ as a reducing agent can lead to pipeline corrosion, obstructions, ammonia leaks and other secondary pollution issues (Martin et al., 2006; Mrad et al., 2015). Selective catalytic reduction of NO with methane (CH_4_-SCR) is a promising new DeNO_x_ technique. This method uses CH_4_ as a reductant instead of NH_3_, enabling the synergistic purification of NOx and greenhouse gas CH_4_, thus avoiding many issues associated with NH_3_ (Campa et al., 2021; Wen et al., 2021).

As the primary component of natural gas, CH_4_ is abundant and readily available. However, CH_4_ is the most stable hydrocarbon molecule, which makes it less effective for selective reduction of NOx at low temperatures and more prone to undergo a non-selective catalytic combustion at high temperatures. Therefore, the CH_4_-SCR technology is still meeting many challenges. Among them, developing effective CH_4_-SCR catalysts is critically important. Currently, the two most developed catalytic systems are metal oxides and molecular sieves. Compared to metal oxide catalysts, molecular sieve catalysts have gained more attention due to their rich pore structure, adjustable acidity, and metal-ion exchange ability (Jin et al., 2009).

Catalysts containing Pd, Ag, Ni, In and Co supported by molecular sieve have been investigated in CH_4_-SCR reaction (Lee et al., 2003; Shi et al., 2004; Chupin et al., 2006; Kubacka et al., 2006; Campa et al., 2015; Sazama et al., 2016). Noble metal-based molecular sieve catalysts can activate CH_4_ at lower temperatures and display a broad reaction temperature window (350°C–500° C), but they are expensive and the CH_4_ selectivity decreases rapidly as the temperature rises. Thus, high CH_4_/NO feed ratios are generally required to achieve high NO conversion in CH_4_-SCR reaction catalyzed by the noble metal-based molecular sieve catalysts (Chen et al., 2011; Costilla et al., 2011). Non-precious metal-based molecular sieve catalysts typically require higher reaction temperatures (>500 °C) for good CH_4_-SCR performance. However, they also face issues of CH_4_ susceptibility to non-selective catalytic combustion at high temperatures (Chen et al., 2011; Huang et al., 2018). Therefore, the key challenge is to design metal-based molecular sieve catalysts that can moderately activate CH_4_ at low temperatures, promoting CH_4_-SCR reactions and achieving high NOx conversion rates.

To develop effective CH_4_-SCR catalysts with good low-temperature activity and high CH_4_ selectivity. In this study, a series of M/SSZ-39 catalysts with different metal loadings (M = In, Co, Cu, Fe) were prepared using a simple wet impregnation method on a SSZ-39 molecular sieves. The physicochemical properties of the catalysts were systematically investigated, and their CH_4_-SCR performance was evaluated to assess the impact of various metal and metal loadings. The catalytic evaluation results showed that In/SSZ-39 catalysts performed significantly better performance than Co-, Cu-, and Fe/SSZ-39 catalysts, with enhanced low-temperature DeNOx activity as In loading increased. Characterization results demonstrated that In species were highly dispersed without nanoparticle formation on the xIn/SSZ-39 catalysts prepared with different loadings (x = 1, 2, 3, x represents the In loadings of 1.0 wt%, 2.0 wt% and 3.0 wt%).

## 2 Experimental section

### 2.1 Materials and instruments

SSZ-39 molecular sieve with a Si/Al ratio of 11 was purchased from Dalian Zhongcaptor Molecular Sieve Factory; Indium nitrate tetrahydrate (In(NO_3_)_3_ 4H_2_O), analytically pure, was obtained from Aladdin Reagent; Analytically pure Cobalt nitrate hexahydrate (Co(NO_3_)_2_ 6H_2_O), Copper nitrate trihydrate (Cu(NO_3_)_2_ 3H_2_O) and Iron nitrate hydrate nine (Fe(NO_3_)_3_ 9H_2_O) were purchased from Tianjin Damao Reagent Factory.

Powder X-ray diffraction (XRD) patterns were recorded on a X′Pert PRO-type diffractometer (Panacor) with a nickel-filtered Cu Kα X-ray source at a scanning rate of 5°/min over the range of 5°–50°. N_2_ adsorption-desorption isotherms were recorded at -196° C on a ASAP-2020-type physical adsorptive instrument. The sample (0.05–0.1 g) was degassed in a vacuum at 150 °C for 4 h prior to measurement. The Brunauer-Emmauer-Teller (BET) method was used to calculate the specific surface area of catalyst. The scanning electron microscopy (SEM) images were taken on a FEI-Verios 460L. Transmission electron microscopic (TEM) images were taken on a Tecnai G220 S-twin instrument (FEI Company) with an acceleration voltage of 200 kV. High-angle annular dark field-scanning transmission electron microscopy (HAADF-STEM) was carried out using a JEM-ARM200F instrument. Energy-dispersive X-ray spectroscopic (EDS) images were collected using a JED-2300T instrument. The H_2_ temperature-programmed reduction (H_2_-TPR) test was conducted on a AutoChem 2920-type chemisorptive instrument. The feeding gas of 10% H_2_ + 90% Ar was fed into the reactor with 60 mL/min, and the H_2_ intensity profiles were record from 50°C to 800° C at a heating rate of 10° C/min.

### 2.2 Preparation of catalysts

The In/SSZ-39 catalysts were prepared using the wet impregnation method with SSZ-39 molecular sieves as the carrier and In(NO_3_)_3_ 4H_2_O as the indium source. Firstly, a suitable amount of In(NO_3_)_3_· 4H_2_O was fully dissolved in deionized water. Then the SSZ-39 molecular sieve was added, and the mixture was agitated for 4 h at room temperature until the mixture slowly evaporated at 80° C. The obtained viscous sample was put into a blast drying oven at 120° C for 12 h. Subsequently, the sample was calcined at 550° C for 4 h with an increase rate of 5° C/min in a muffle furnace. The obtained catalysts were labeled as xIn/SSZ-39, where x represents the theoretical In loadings of 1.0 wt%, 2.0 wt%, and 3.0 wt%, respectively.

The xCo/SSZ-39, xCu/SSZ-39 and xFe/SSZ-39 catalysts were prepared using the same method as that of the xIn/SSZ-39 catalysts, except that In(NO_3_)_3_ 4H_2_O was replaced by Co(NO_3_)_2_ 6H_2_O, Cu(NO_3_)_2_ 3H_2_O, and Fe(NO_3_)_3_ 9H_2_O, respectively, and x represents the theoretical metal loading 3.0 wt%.

### 2.3 Evaluation of the catalyst activities

Catalytic activity tests were conducted in a fixed-bed reactor at atmosphere pressure. A total of 0.75 mL catalyst with a particle size of 40–60 mesh was filled in a fixed-bed reactor with an inner diameter of 8 mm. The feed gas consisted of 0.1 vol% NO, 0.1 vol% CH_4_, 6 vol% O_2_ and balance N_2_, with a GHSV of 8,000 h^−1^. Before the measurement, the catalyst was pretreated at 400° C for 30 min under N_2_ atmosphere to remove any adsorbed impurity gases and moisture on the catalyst. During tests, the steady-state outlet compositions (NO, NO_2_, N_2_O and CH_4_ concentrations) at different reaction temperature (400, 450, 500°C and 550°C) were measured by an on-line infrared flue gas analyzer (MKS Instruments, Inc., 6030). The NO conversion rate, CH_4_ conversion rate and CH_4_ selectivity were calculated using Eqs 1–3, respectively, as shown below:

$$C_{NO}=\frac{{\emptyset \left(NO\right)}_{in}-{\emptyset \left(NO\right)}_{out}{-\emptyset \left({NO}_{2}\right)}_{out}}{{\emptyset \left(NO\right)}_{in}}\times 100\%$$ (1)

$$C_{{CH}_{4}}=\frac{{\emptyset \left({CH}_{4}\right)}_{in}-{\emptyset \left({CH}_{4}\right)}_{out}}{{\emptyset \left({CH}_{4}\right)}_{in}}\times 100\%$$ (2)

$$S_{{CH}_{4}}=\frac{{\emptyset \left(NO\right)}_{in}-{\emptyset \left(NO\right)}_{out}{-\emptyset \left({NO}_{2}\right)}_{out}}{2\left[{\emptyset \left({CH}_{4}\right)}_{in}-{\emptyset \left({CH}_{4}\right)}_{out}\right]}\times 100\%$$ (3)

where $C_{NO}$ and $C_{{CH}_{4}}$ are the NO and CH_4_ conversion rates, respectively. ${\emptyset \left(NO\right)}_{in}$ , ${\emptyset \left(NO\right)}_{out}$ , ${\emptyset \left({CH}_{4}\right)}_{in}$ and ${\emptyset \left({CH}_{4}\right)}_{out}$ are the concentrations of NO and CH_4_ at the reactor inlet and outlet, respectively. ${\emptyset \left({NO}_{2}\right)}_{out}$ is the NO_2_ concentration at the reactor outlet. $S_{{CH}_{4}}$ is the CH_4_ selectivity.

## 3 Results and discussion

### 3.1 Characterization results of catalysts

**FIGURE 1:**
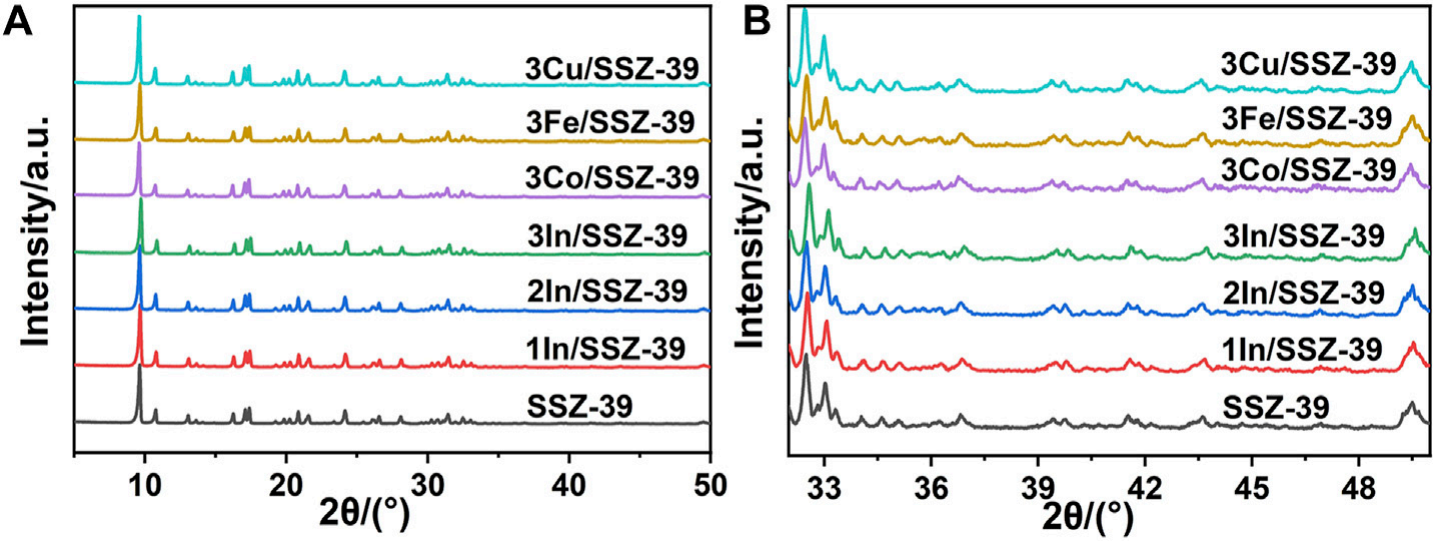
XRD patterns **(A)** and corresponding partial enlarged patterns **(B)** of different catalysts.

Figure 1 presents the XRD spectra of M/SSZ-39 catalysts. A series of distinctive diffraction peaks at 2θ = 9°–11° and 2θ = 15°–18° are present in all samples, which are attributed to the typical AEI topology of SSZ-39 molecular sieves (Li et al., 2019; Du et al., 2023). The XRD spectra of M/SSZ-39 catalysts with different metals and different metal loadings do not change significantly, indicating that the loading of metals through the wet impregnation procedure does not affect the AEI topology of SSZ-39 molecular sieve. Figure 1B shows a localized enlarged XRD spectrum of Figure 1A, where no diffraction peaks of metal oxides are observed in all catalysts. This indicates that metal species exist as highly dispersed metal oxide clusters or ionic metal sites on SSZ-39 molecular sieve at higher metal loading. This phenomenon is attributed to the large specific surface area and regular cage structure of the small-pore SSZ-39 molecular sieve, which provides a lot of space and specific surface for dispersing metal species (Ren et al., 2011; Lim et al., 2020; Xu et al., 2022).

**FIGURE 2:**
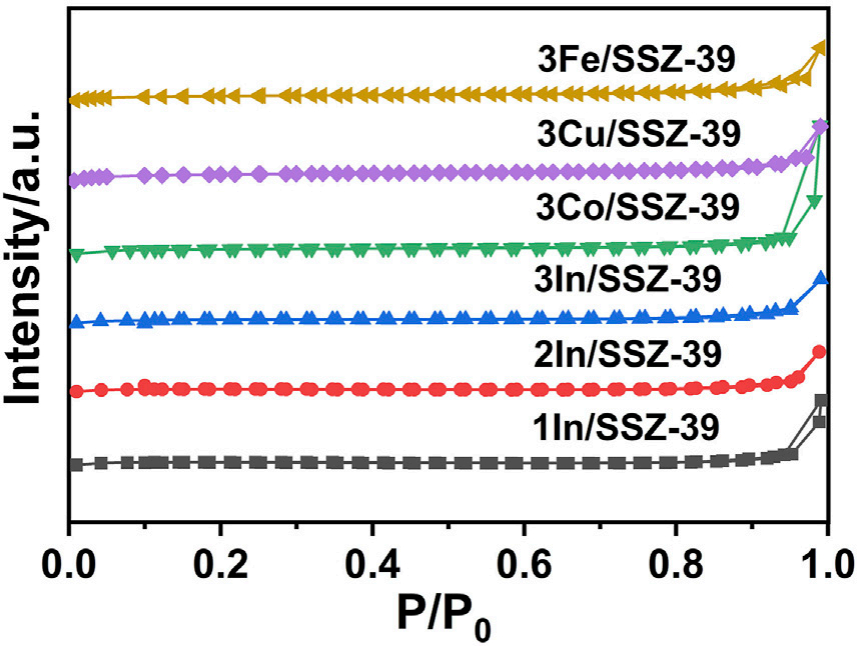
N_2_ adsorption-desorption isotherms of different catalysts.

The textual properties of xIn/SSZ-39 catalysts were characterized by N_2_ adsorption-desorption isotherms, as shown in Figure 2. All samples display type I adsorption isotherm features, typical adsorption isotherm characteristics of microporous materials (Kruk et al., 2001). This is consistent with the uniform microporous nature of small-pore SSZ-39 molecular sieve. Table 1 shows the calculated textual properties of xIn/SSZ-39. With increasing In loading, all catalystsʹ specific surface areas only slightly decreased. Notably, the 1In/SSZ-39 and 2In/SSZ-39 catalysts exhibit nearly identical microporous specific surface area, exterior specific surface area, and microporous pore volume. This demonstrates that the In species are able to maintain a high degree of dispersion and do not obstruct the microporous pores of SSZ-39 when the loading of In is controlled to be no more than 2 wt%. When the In loading is increased to 3 wt%, the microporous specific surface area of 3In/SSZ-39 catalyst begins to decrease slightly, while its external specific surface area became significantly larger. This may be attributed to the formation of highly dispersed InOx clusters on the external surface of SSZ-39, which increases the external specific surface area of 3In/SSZ-39 catalyst (no InOx nanoparticles were observed in the TEM images). The specific surface area of 3Co/SSZ-39, 3Cu/SSZ-39, and 3Fe/SSZ-39 catalysts are similar to that of In/SSZ-39 catalyst. It indicates that these metal species (Co, Cu and Fe) are also highly dispersed on SSZ-39.

**TABLE 1:** Textual properties of differen catalysts.

Samples	S_BET_ (m^2^/g)			Pore volume (cm^3^/g)	
	S_total_ ^a^	S_micro_ ^b^	S_ext_ ^c^	V_total_	V_micro_ ^d^
1In/SSZ-39	519.76	518.92	0.84	0.27	0.26
2In/SSZ-39	518.99	517.76	1.23	0.27	0.26
3In/SSZ-39	507.49	500.85	6.64	0.27	0.25
3Co/SSZ-39	523.15	518.16	4.99	0.27	0.26
3Cu/SSZ-39	502.72	495.54	7.18	0.27	0.25
3Fe/SSZ-39	506.35	500.18	6.17	0.27	0.25

^a^ BET, surface area.

^b^ t-plot micropore surface area.

^c^ t-plot external surface area.

^d^ t-plot micropore volume.

**FIGURE 3:**
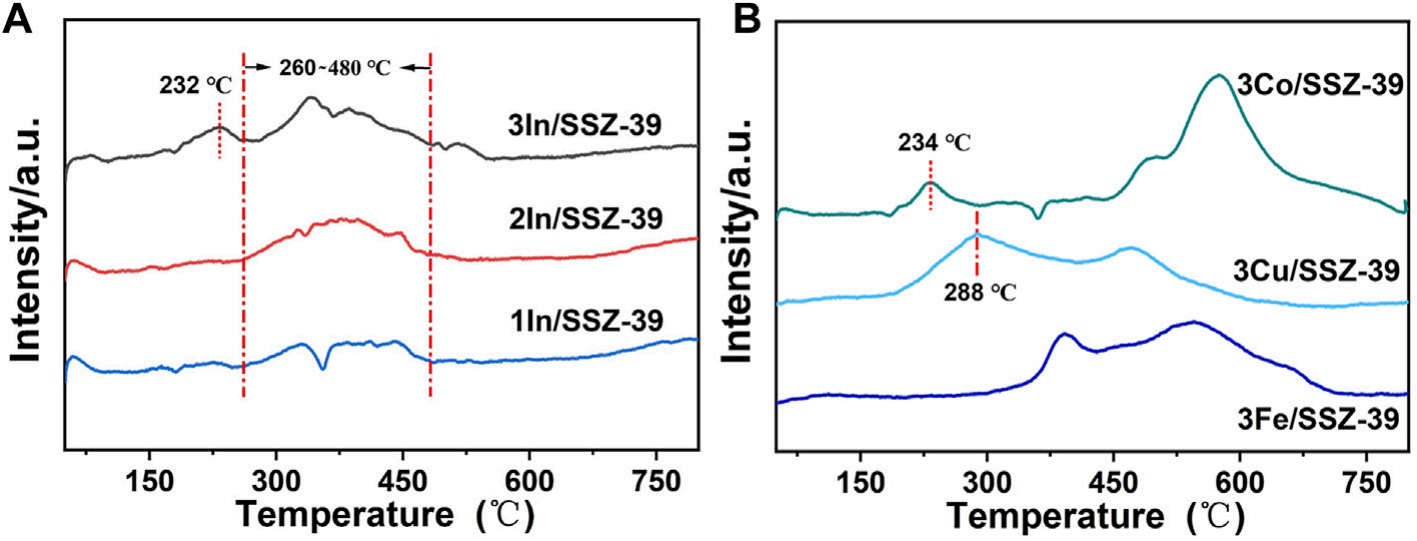
H2-TPR profiles of **(A)** xIn/SSZ-39 catalysts and **(B)** 3Co/SSZ-39, 3Cu/SSZ-39 and 3Fe/SSZ-39 catalysts.

The redox properties of catalyst are crucial for CH_4_-SCR activity, therefore, H_2_-TPR characterization was carried out to investigate the reducibility of metal oxide species in catalysts, as shown in Figure 3. In Figure 3A, it can be observed that the H_2_-TPR spectra of all In/SSZ-39 catalysts display broad reduction signals between 260° C and 480° C, which are attributed to the reduction of InOx species to In^+^ sites (Yoo et al., 2015; Zhao et al., 2023). Moreover, the primary reduction peaks of three catalysts are almost located in the same positions, centered at 260°C–480° C, without shifting to lower or higher temperature regions. This demonstrates that the forms of the major InOx species in catalysts are essentially the same. The H_2_ consumption gradually increased as In loading increased, corresponding to the increased area of the major reduction peaks in xIn/SSZ-39 catalysts. Remarkably, a notable reduction peak centered at a lower temperature of only ∼232 °C appeared in the H_2_-TPR profile of 3In/SSZ-39 catalyst, suggesting the better reducibility of highly dispersed InOx species. This phenomenon can be attributed to the formation of highly dispersed InOx clusters on the external surface of SSZ-39 resulting from the increased In loading, as corroborated by the N_2_ physical adsorption-desorption test. This fraction of the InOx species exhibits better reducibility as it is not constrained by the pores or cavities of SSZ-39, which is essential for the moderate activation of CH_4_ and contributes significantly to the enhancement of low-temperature CH_4_-SCR performance (Yoo et al., 2015; Zhao et al., 2023).

In Figure 3B, the 3Co/SSZ-39, 3Cu/SSZ-39 and 3Fe/SSZ-39 catalysts display a significantly different H_2_-TPR spectrum compared to the 3In/SSZ-39 catalyst. A primary reduction peak is located at a lower temperature of 288° C in 3Cu/SSZ-39 catalyst which is much lower than that of 3Co/SSZ-39, 3Fe/SSZ-39 and 3In/SSZ-39 catalysts, suggesting the good reducibility of major CuOx species. This is conducive to activating CH_4_ at low-temperatures. However, the major CuOx species, with good reducibility, also facilitates the non-selective catalytic combustion reaction, may leading to very low N_2_ selectivity of 3Cu/SSZ-39 catalyst. In the H_2_-TPR spectrum of 3Fe/SSZ-39 catalyst, there are not any reduction peaks located at lower temperatures can be observed, and the major reduction peaks are almost located at higher temperatures between 375°C and 700° C. The results suggest that FeOx species are not conducive to the activation of CH_4_ at low temperatures. For 3Co/SSZ-39 catalysts, a small reduction peak centered at 234 °C appeared in the H_2_-TPR profile similar to that of 3In/SSZ-39. But, the major reduction peak located at higher temperatures between 450°C and 700° C which are much higher than that (260°C–480° C) of 3In/SSZ-39 catalysts. In the H_2_-TPR spectrum of 3In/SSZ-39 catalyst, a small reduction peak located at lower temperature of 232° C and a major reduction peak located at medium temperature of 342° C are observed, indicating the moderate reducibility of highly dispersed InOx species. Compared to the 3Co/SSZ-39, 3Cu/SSZ-39 and 3Fe/SSZ-39 catalysts, the InOx species in 3In/SSZ-39 catalyst, with moderate reducibility, may promote the moderate activation of CH_4_ and enhance the CH_4_-SCR activity at low temperatures.

**FIGURE 4:**
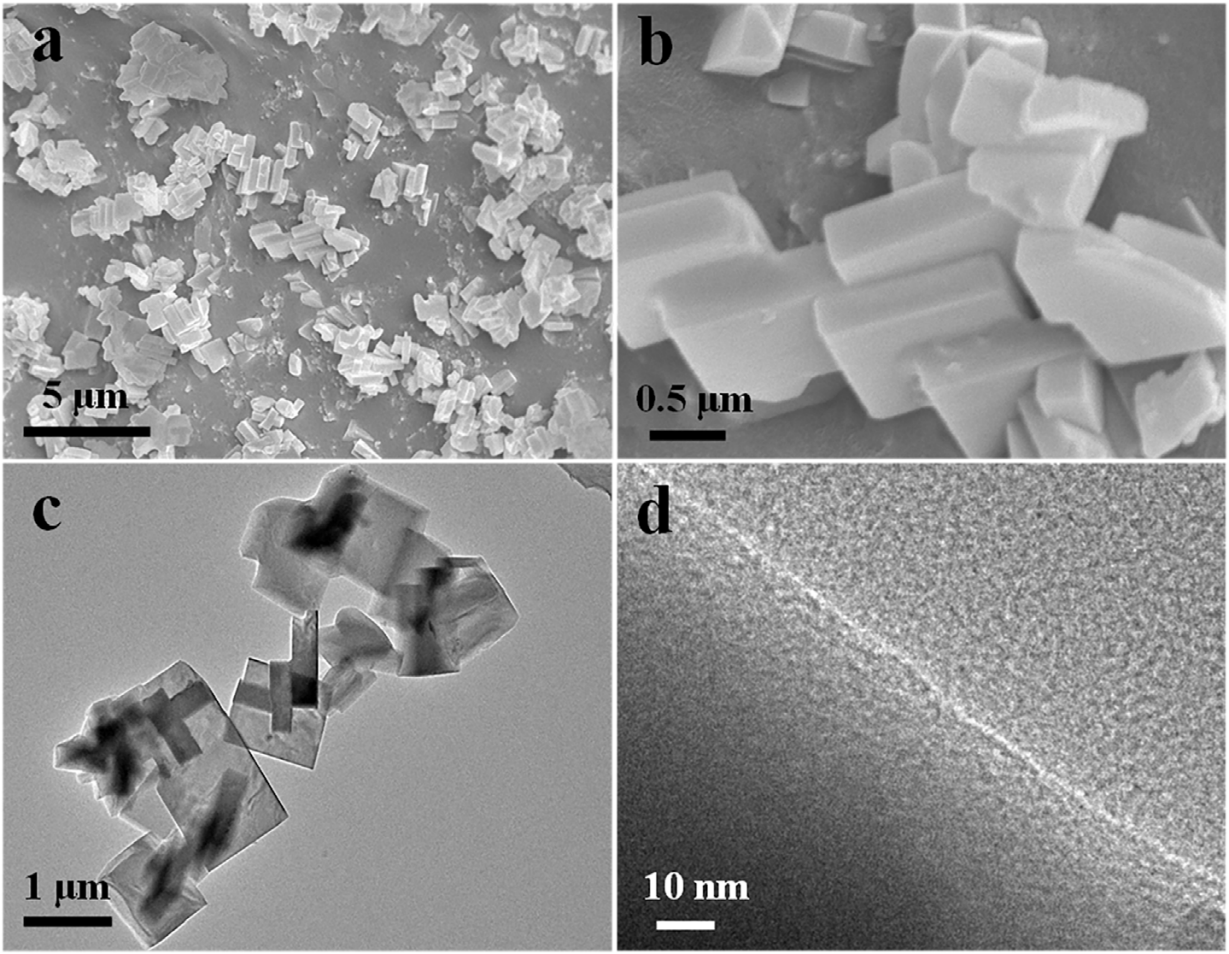
SEM **(A, B)**, TEM **(C)** and HRTEM **(D)** images of 3In/SSZ-39.

**FIGURE 5:**
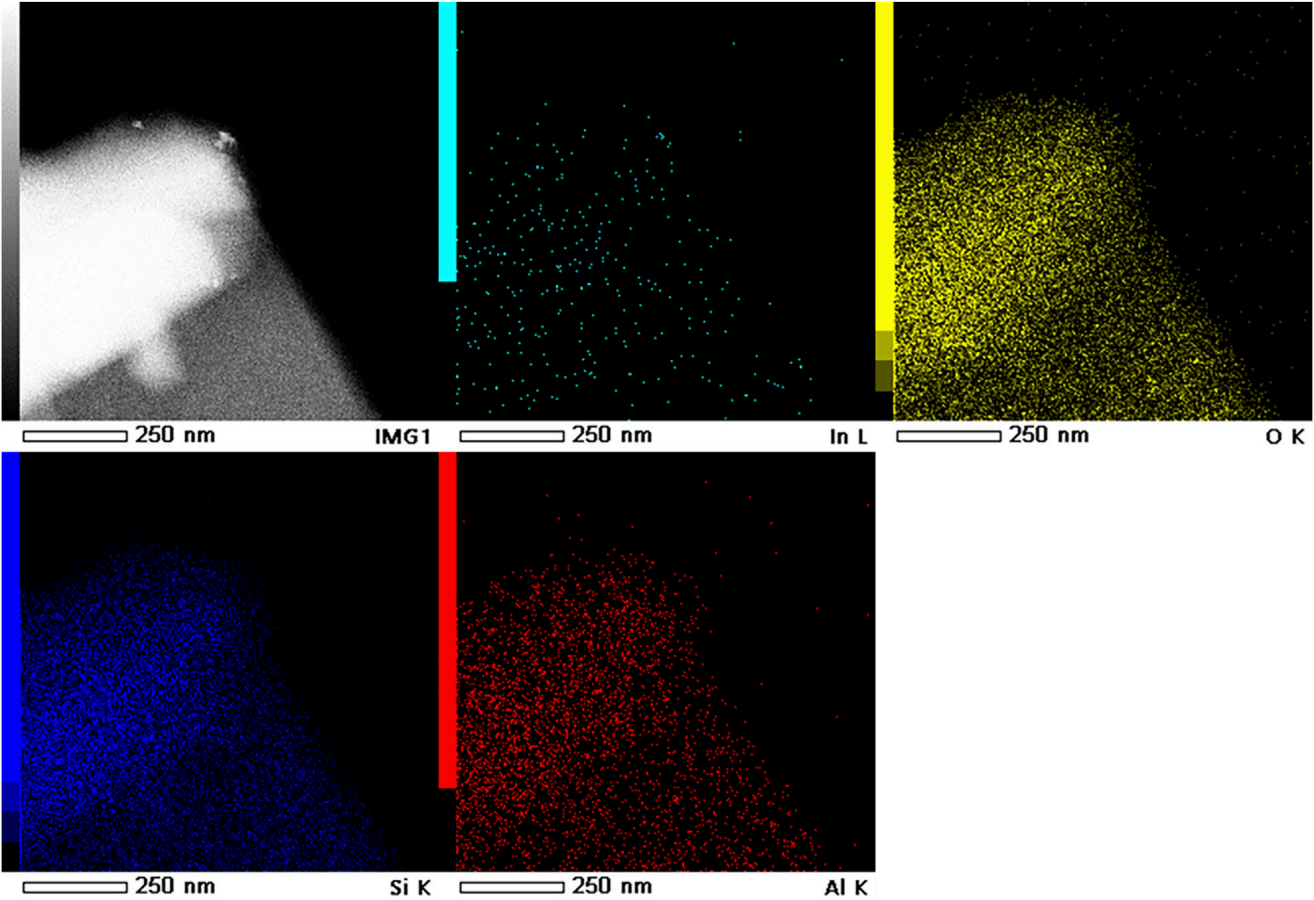
HAADF-STEM image and corresponding element mappings of 3In/SSZ-39.

Figures 4A,B depict the SEM images of 3In/SSZ-39 catalyst, revealing typical zeolite particles with a regular and smooth hexahedral structure, and no nanoparticles are observed on the surface of the smooth hexahedral structure. Further, TEM and EDS characterizations were conducted to examine the dispersion form of InOx species on SSZ-39 molecular sieve, as shown in Figure 4C, 4days and Figure 5. The basic hexahedral structure of SSZ-39 remains intact, consistent with the SEM results. Figure 4D presents the high-resolution TEM image of 3In/SSZ-39 catalyst, where no distinct InOx nanoparticles were observed, aligning with the XRD results. The combination of N_2_ physical adsorption-desorption tests and H_2_-TPR characterizations strongly indicates that the InOx species are highly dispersed on the SSZ-39 molecular sieve, possibly existing in the form of InOx clusters or In^+^ sites, and do not aggregate to form nanoparticles. This hypothesis is further supported by the EDS characterization, which shows that no InOx nanoparticles are generated since the In elements are extremely uniformly dispersed in SSZ-39 molecular sieves (Figure 5).

### 3.2 CH_4_-SCR performance of catalysts

**FIGURE 6:**
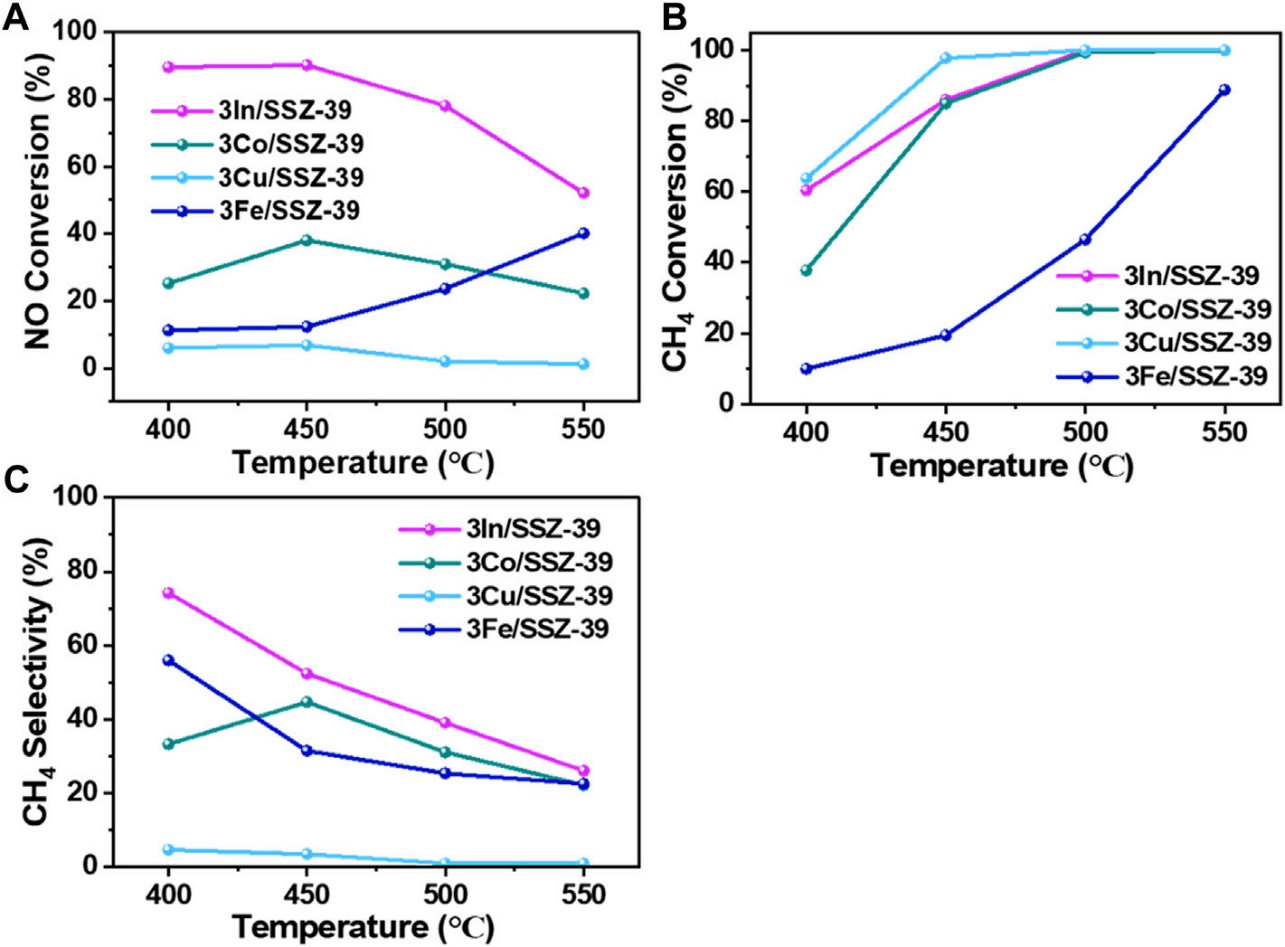
NO conversion rates **(A)**, CH_4_ conversion rates **(B)** and CH_4_ selectivities **(C)** as a function of temperatures in CH_4_-SCR over 3In/SSZ-39, 3Co/SSZ-39, 3Cu/SSZ-39 and 3Fe/SSZ-39 catalysts (NO/CH_4_ = 1).

The CH_4_-SCR performance of M/SSZ-39 catalysts (40–60 mesh) was tested in a fixed-bed reactor at 400°C–550° C, using a feed gas composed of 0.1 vol% NO, 0.1 vol% CH_4_, 6 vol% O_2_ and the balance N_2_ with a GHSV of 8,000 h^−1^. Initially, the effects of different metal components on the performance of catalysts for CH_4_-SCR were investigated. Figure 6A displays the temperature-dependent NO conversion curves of 3In/SSZ-39, 3Co/SSZ-39, 3Cu/SSZ-39, and 3Fe/SSZ-39 catalysts. It is evident that the activities of catalysts follow the order: In > Co > Fe > Cu when the reaction temperature is below 530° C. Notably, the 3In/SSZ-39 catalyst exhibits significant performance compared to the 3Co, 3Cu, and 3Fe/SSZ-39 catalysts, underscoring the significance of In as an active component in catalyzing the selective catalytic reduction of NOx by CH_4_ (Zhao et al., 2023). As indicated by the H_2_-TPR results, compared to the 3Co/SSZ-39, 3Cu/SSZ-39 and 3Fe/SSZ-39 catalysts, the highly dispersed InOx species in 3In/SSZ-39 catalyst, with moderate reducibility, can promote the moderate activation of CH_4_ and enhance the SCR process at low temperatures. Therefore, the 3In/SSZ-39 catalyst exhibits excellent CH_4_-SCR activity and good CH_4_ selectivity, achieving a high NO conversion rate over 90% at lower temperatures (400°C–450° C) and a lower CH_4_/NO feed ratios (CH_4_/NO = 1/1).

The NO conversion rate of 3In/SSZ-39 catalyst remains almost unchanged at 90% (Figure 6A) as the reaction temperature is increased from 400°C to 450 °C. However, as shown in Figure 6B, the CH_4_ conversion rate increased from 60% to 86%. This suggests that 26% of the converted CH_4_ undergoes non-selective catalytic combustion rather than a selective reduction of NOx, demonstrating that the 3In/SSZ-39 catalyst achieves the highest CH_4_ selectivity and NO conversion rate at 400 °C, as depicted in Figure 6C. In contrast, compared to NO conversion rates, the CH_4_ conversion rates of the 3Co/SSZ-39 and 3Cu/SSZ-39 catalysts are significantly higher, suggesting that the Co and Cu components are more prone to catalyzing the CH_4_ combustion reaction, leading to lower CH_4_ selectivity, as illustrated in Figure 6C. As for the 3Fe/SSZ-39 catalyst, both lower NO and CH_4_ conversion rates are achieved, indicating that the Fe component is less effective in catalyzing CH_4_ catalytic combustion and CH_4_-SCR reaction. In conclusion, the types of metal components can be tuned to control the competition between CH_4_ non-selective catalytic combustion and CH_4_-SCR reactions.

**FIGURE 7:**
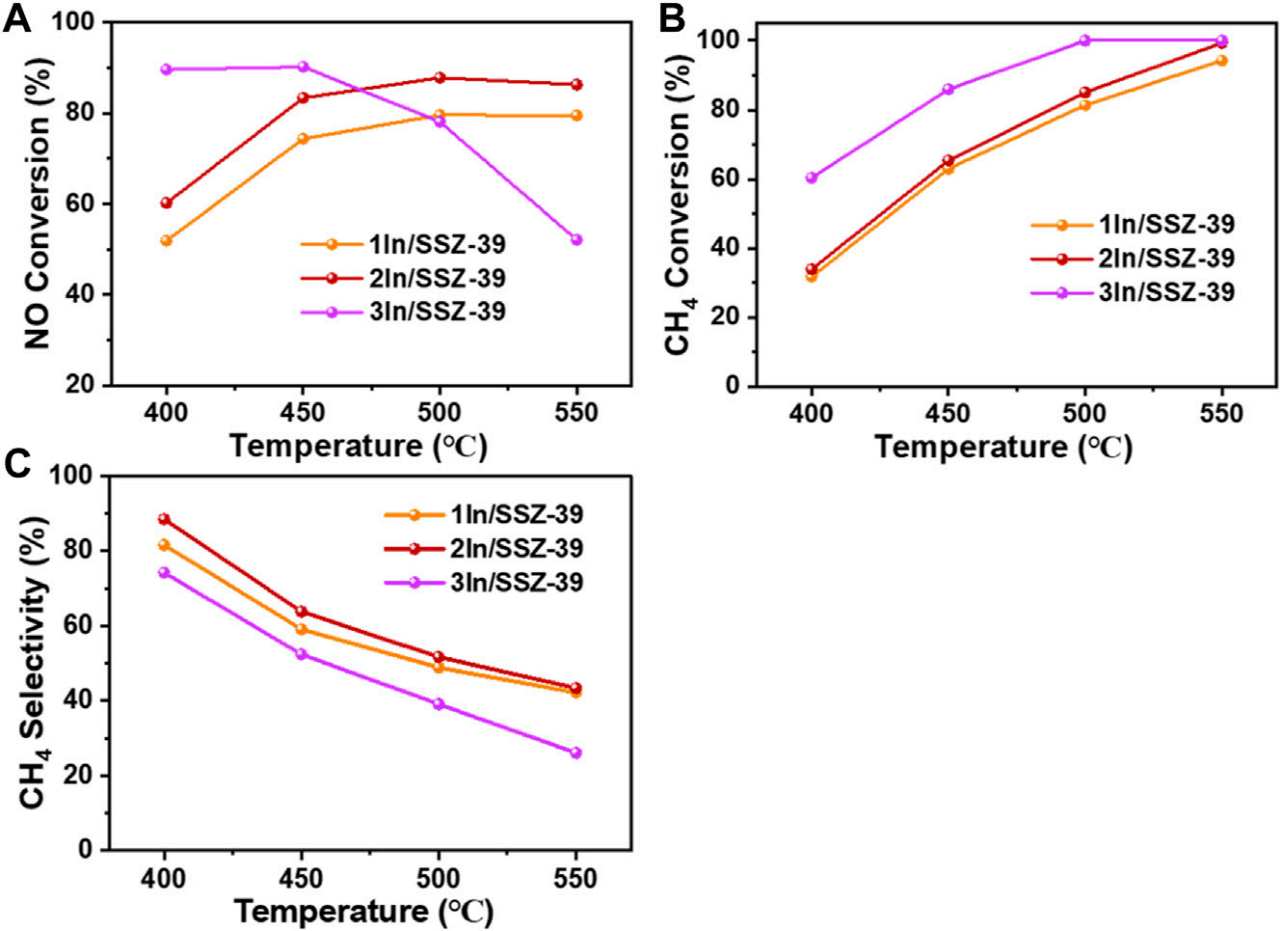
NO conversion rates **(A)** CH_4_ conversion rates **(B)** and CH_4_ selectivities **(C)** as a function of temperatures in CH_4_-SCR over 1In/SSZ-39, 2In/SSZ-39 and 3In/SSZ-39 catalysts (NO/CH_4_ = 1).

For the xIn/SSZ-39 catalysts with superior CH_4_-SCR performance, the impact of various In loadings on the catalytic performance was further investigated, as shown in Figure 7. It is evident that both the NO and CH_4_ conversion rates of catalysts increase with temperature when the in loading is less than 2 wt%, corresponding to their high CH_4_ selectivity, as illustrated in Figure 7C. Among these, the 2In/SSZ-39 catalyst exhibits a 60% NO conversion rate and a 34% CH_4_ conversion rate at 400° C, indicating that the ratio of converted NO to CH_4_ molecules is close to 2, which is the theoretical NO/CH_4_ ratio when a reaction proceeds in accordance with the stoichiometric ratio. This demonstrates that the 2In/SSZ-39 catalyst can achieve a very high CH_4_ selectivity (up to 88.5%) at 400° C, as shown in Figure 7C. For the 3In/SSZ-39 catalyst, it is NO conversion rate decreases rapidly as the temperature raised from 450°C to 550° C, indicating a rapid decrease in CH_4_ selectivity as well. This can be attributed to the presence of highly dispersed InOx clusters on the external surface of SSZ-39 (as indicated by H_2_-TPR results). These clusters exhibit the best reducibility (low-temperature reduction peak of only ∼232° C), which promote the moderate activation of CH_4_ and enhance the CH_4_-SCR activity at low temperatures. However, at higher temperatures, these highly active InOx clusters can lead to over-activation of CH_4_, which increases the likelihood of converted CH_4_ undergoing a non-selective catalytic combustion reaction and thus reducing the CH_4_ selectivity.

## 4 Conclusion

In this paper, a series of M/SSZ-39 catalysts (M = In, Co, Cu, Fe) were prepared using the wet impregnation method on a small-pore SSZ-39 molecular sieve. The effects of different metal loadings and metal loadings on the performance of CH_4_-SCR were investigated. The characterization results revealed that the performance of In/SSZ-39 catalysts are significantly higher than that of the Cu-, Co-, and Fe/SSZ-39 catalysts, indicating that indium (In) is a more suitable active ingredient for the CH_4_-SCR reaction. The low-temperature CH_4_-SCR activity of catalysts is notably enhanced with increasing In loadings. Among them, the 3In/SSZ-39 catalyst exhibits a high NO conversion rate of up to 90% and a CH_4_ selectivity of up to 74.2% at a reaction temperature of 400 °C and a low CH_4_/NO feed ratio (CH_4_/NO = 1). TEM and EDS characterization results showed that the In species were highly dispersed on the SSZ-39 molecular sieves, greatly promoting the moderate activation of CH_4_ and enhancing the CH_4_-SCR activity at low temperatures. These findings demonstrate a potentially generalizable approach toward the design and synthesis of high-efficiency CH_4_-SCR catalyst.

## Data Availability

The original contributions presented in the study are included in the article/Supplementary Material, further inquiries can be directed to the corresponding authors.
